# Mitochondrial dysfunction in inflammatory bowel disease

**DOI:** 10.3389/fcell.2015.00062

**Published:** 2015-10-01

**Authors:** Elizabeth A. Novak, Kevin P. Mollen

**Affiliations:** Department of Surgery, University of Pittsburgh School of MedicinePittsburgh, PA, USA

**Keywords:** mitochondrial dysfunction, inflammatory bowel disease, intestinal inflammation, metabolic stress, reactive oxygen species, inflammasome, gut-barrier function, autophagy

## Abstract

Inflammatory Bowel Disease (IBD) represents a group of idiopathic disorders characterized by chronic or recurring inflammation of the gastrointestinal tract. While the exact etiology of disease is unknown, IBD is recognized to be a complex, multifactorial disease that results from an intricate interplay of genetic predisposition, an altered immune response, changes in the intestinal microbiota, and environmental factors. Together, these contribute to a destruction of the intestinal epithelial barrier, increased gut permeability, and an influx of immune cells. Given that most cellular functions as well as maintenance of the epithelial barrier is energy-dependent, it is logical to assume that mitochondrial dysfunction may play a key role in both the onset and recurrence of disease. Indeed several studies have demonstrated evidence of mitochondrial stress and alterations in mitochondrial function within the intestinal epithelium of patients with IBD and mice undergoing experimental colitis. Although the hallmarks of mitochondrial dysfunction, including oxidative stress and impaired ATP production are known to be evident in the intestines of patients with IBD, it is as yet unclear whether these processes occur as a cause of consequence of disease. We provide a current review of mitochondrial function in the setting of intestinal inflammation during IBD.

## Introduction: inflammatory bowel disease

Inflammatory bowel disease (IBD) is a complex, chronic, relapsing, and remitting inflammatory condition of the gastrointestinal tract characterized by symptoms such as diarrhea, bloody stools, abdominal pain, and weight loss (Greco et al., 2011; Indriolo et al., 2011; Rigoli and Caruso, 2014). There are two diseases which fall under the heading of IBD: Crohn's disease and ulcerative colitis. Crohn's disease is characterized by transmural inflammation that may affect any part of the gastrointestinal tract, and presentation of disease is dependent upon both location and severity of inflammation (Podolsky, 2002; Indriolo et al., 2011). Inflammation in ulcerative colitis is limited to the mucosa of the colon and rectum. The pattern of clinical disease in IBD is often cyclical with periods of active inflammation and subsequent remissions (Indriolo et al., 2011). Additionally, there is a strong association between IBD and development of colorectal cancer (Persson et al., 1994; Canavan et al., 2006; Grivennikov, 2013). Although there is no cure for IBD, a range of therapeutics (e.g., corticosteroids, immunomodulators, antibiotics, aminosalicylates, and biologic therapies) is employed to help manage the symptoms of disease. The results of medical treatment are highly variable, and the potential exists for significant morbidity over a long lifetime with disease.

In the United States, it is currently estimated that approximately 1.6 million people suffer from IBD, with as many as 70,000 new cases reported each year (Crohn's and Colitis Foundation of America, 2014). The peak age of onset is between 15 and 35 years with approximately 5–10% of patients diagnosed during childhood (< 20 years). IBD is a chronic, lifelong disease that creates a vast financial burden. Previous studies have estimated that the annual direct health care cost for a patient with Crohn's disease is $8265–18,963 and $5066–15,020 per patient diagnosed with ulcerative colitis (Gibson et al., 2008). Extrapolating these figures onto the current prevalence estimates of IBD reveals an estimated annual total direct cost of between 11 and 28 billion dollars (Crohn's and Colitis Foundation of America, 2014).

While the exact pathophysiology of IBD is not yet understood, it is known that the disease is triggered by a complex interaction between genetic, environmental, and immunoregulatory factors. Studies have delineated a clear genetic link to disease. Children of parents affected by IBD have an increased risk of developing the disease (Noble and Arnott, 2008). The risk is significantly higher when both parents have IBD, with disease developing in up to 36% of people with two parents previously diagnosed with IBD (Bennett et al., 1991). Other studies have demonstrated a much higher disease frequency (5–20% increase) amongst first-degree relatives of affected individuals compared to the general population (Russell and Satsangi, 2008). The familial link appears to be stronger in Crohn's disease compared to ulcerative colitis (Tysk et al., 1988; Thompson et al., 1996; Orholm et al., 2000; Halfvarson et al., 2003). Although genetics clearly play a role in disease, the exact nature of genetic predisposition is quite complex, and it is possible that susceptibility to IBD may involve the interaction of several genes. To date, genome-wide association studies (GWAS) have identified more than 160 genetic loci that confer susceptibility to disease (Liu et al., 2015). The fact that genetic polymorphism alone does not predict disease, but merely confers risk of developing IBD highlights the importance that other elements, such as environmental constituents, must also be a contributing factor (Cho and Brant, 2011). Interestingly, the majority of the genetic loci confer susceptibility to both ulcerative colitis and Crohn's disease, calling into question the rigid categorization of IBD subsets.

Although the precise environmental factors that trigger IBD are not known, several risk factors, including antibiotic exposure, stress, dysbiosis, and nonsteroidal anti-inflammatory drug exposure (NSAIDs), are thought to play a role in disease onset and progression (Loftus, 2004; Bernstein et al., 2006; Bernstein, 2008; Molodecky and Kaplan, 2010). Research has demonstrated that IBD is more common in developed countries compared to developing countries, which suggests that many factors associated with the “westernized” lifestyle, such as diet, decreased exposure to sunlight, exposure to pollution and industrial chemicals, may be associated with disease development (Hanauer, 2006). Interestingly, the incidence of IBD in some developing countries (e.g., India and China) is beginning to rise as they become more industrialized (Desai and Gupte, 2005; Zheng et al., 2005). Likewise, migrant studies have revealed that when populations relocate from regions of low IBD prevalence to regions of higher prevalence, they acquire an increased risk of developing disease (Bernstein and Shanahan, 2008; Mikhailov and Furner, 2009). This highlights the importance of environmental factors in the onset and progression of disease in susceptible hosts (Hanauer, 2006).

An evolving body of literature would suggest that predisposing factors converge, resulting in a breakdown of the intestinal barrier and the translocation of luminal antigens. In genetically susceptible individuals, this bacterial translocation triggers a dysfunctional mucosal immune response and promotes inflammation. Although the theory of increased intestinal epithelial permeability as a primary cause of IBD has yet to be proven, it is supported by murine models of experimental colitis (Madsen et al., 1999; Resta-Lenert et al., 2005; Turner, 2009) and some human studies (Söderholm et al., 1999; Zeissig et al., 2007). Since the maintenance of epithelial junction integrity is energy-dependent, it would suggest that mitochondrial function might be central for the appropriate preservation of epithelial barrier function. Interestingly, constituents that have the potential to contribute to IBD susceptibility, such as gastrointestinal infection, and nonsteroidal anti-inflammatory drugs, have also been shown to affect mitochondrial function (Roediger, 1980a; Singh et al., 2009b; Schoultz et al., 2011). Additionally, structurally abnormal mitochondria have been observed in both animal models of intestinal disease (Rodenburg et al., 2008) and in tissues from patients with intestinal inflammation (Nazli et al., 2004). Moreover, processes which influence mitochondrial function, such as autophagy (Travassos et al., 2010), endoplasmic reticulum (ER) stress (Kaser et al., 2008), and the dysregulated production of reactive oxygen species (ROS) (Pavlick et al., 2002; Restivo et al., 2004; Beltrán et al., 2010) have all been implicated in IBD. Despite the present interest in mitochondrial function in the pathophysiology of diabetes (Chowdhury et al., 2013), obesity (Rath and Haller, 2011), and neuromuscular disease (Tarnopolsky and Raha, 2005), little is known about the biological behavior of mitochondria in intestinal inflammation. Here, we summarize the current literature that implicates mitochondrial dysfunction in the pathogenesis of IBD.

## Mitochondrial homeostasis

Mitochondria are membrane-bound organelles that maintain cellular energy production through oxidative phosphorylation (Mitchell and Moyle, 1967). Mitochondria contain a circular genome that encodes 13 proteins and the 22 tRNAs and 2 rRNAs needed to translate those proteins within the mitochondrial matrix. All 13 proteins encoded by mitochondrial DNA (mtDNA) form essential subunits of the respiratory complexes I, III, IV, and V (Anderson et al., 1981; Taanman, 1999). The small mitochondrial genome necessitates that nuclear-encoded genes provide the majority of proteins required for the respiratory apparatus as well as all of the enzymes involved in other cellular biosynthetic and oxidative functions (Anderson et al., 1981; Taanman, 1999). Despite the limited coding-capacity of the mtDNA, mitochondria regulate vital cellular functions aside from energy production, such as the generation of ROS and reactive nitrogen species (RNS), the induction of programmed cell death, and the transduction of stress and metabolic signals (Galluzzi et al., 2012; Tait and Green, 2012). Thus, it is necessary that mitochondrial dynamics be tightly controlled in order to maintain overall cellular homeostasis.

Mitochondrial biogenesis, the process by which new mitochondria are generated and repaired, plays a significant role in maintaining cellular metabolic homeostasis. Through the growth and division of established mitochondria, the transcription and assembly of new mitochondrial proteins, or the *de novo* synthesis of new mitochondria, mitochondrial biogenesis provides the cell with an adequate pool of healthy mitochondria. This process is influenced by numerous cellular environmental stresses, such as caloric restriction, hypothermia, exercise, cell division, and oxidative stress (Wenz, 2013). Variations in mitochondrial number, size, and mass exist between all cells and are reflective of the current cellular metabolic state (Leary et al., 1998; Leverve and Fontaine, 2001; Pfeiffer et al., 2001; Kunz, 2003). Mitochondrial biogenesis is a complex process, utilizing mitochondrial proteins encoded by both the mitochondrial and nuclear genomes; thus, precise communication between the mitochondria and nucleus is extremely important. Peroxisome proliferator-activated receptor gamma coactivator 1-α (PGC1-α) is a co-transcriptional regulation factor that is a central modulator of mitochondrial biogenesis (Puigserver et al., 1998). It drives biogenesis by activating various transcription factors, such as nuclear respiratory factor-1 (NRF-1) and nuclear respiratory factor-2 (NRF-2), which not only control the expression of nuclear genes that encode mitochondrial proteins, but also interact with mitochondrial transcription factor A (Tfam) (Jornayvaz and Shulman, 2010), which promotes the transcription and replication of the mitochondrial genome (Virbasius and Scarpulla, 1994).

The competing processes of mitochondrial fusion and fission operate to preserve mitochondrial function or eliminate irreparably damaged mitochondria, respectively. Through their role in regulating mitochondrial dynamics, fusion and fission events fine-tune biological processes central to cell survival, such as ATP generation, calcium homeostasis, and ROS generation. Consequently, they also play a role in apoptosis, mitophagy, cell-cycle progression, and oxygen sensing (Archer, 2013). Highly conserved guanosine triphosphates (GTPases) regulate both processes of fusion and fission (Youle and van der Bliek, 2012; Ishihara et al., 2013). Fusion is regulated by isoforms of two proteins in the outer mitochondrial membrane (OMM), mitofusion-1 and mitofusion-2, and by a dynamin family member, optic atrophy 1 (Opa1) protein, in the inner mitochondrial membrane (IMM) (Youle and van der Bliek, 2012). Mitofusions initiate fusion between neighboring mitochondria through the formation of homodimeric or heterodimeric linkages (Santel and Fuller, 2001; Chen et al., 2003; Hoppins et al., 2007). Opa1 then facilitates the merging of the IMMs (Alexander et al., 2000; Hoppins et al., 2007). Mitofusion-2 also localizes to the ER, where it alters mitochondrial and ER morphology and encourages ER-mitochondria tethering, which enhances calcium signaling (Rojo et al., 2002; de Brito and Scorrano, 2008). Fusion allows for mitochondrial complementation by permitting two mitochondria to fuse and compensate for the defects of each other, thereby generating all of the compulsory machineries for a functional mitochondrial organelle (Archer, 2013). Mitochondria with mtDNA mutations are allowed to fuse with other mitochondria as long as the total mutation burden remains below 80–90% for the cell (Yoneda et al., 1994; Nakada et al., 2001). Mitochondrial fusion is an attempt to buffer brief stresses and fractional defects through the exchange of components in the matrix and intermembrane space (Nunnari et al., 1997; Ono et al., 2001; Chan, 2006; Youle and van der Bliek, 2012).

When mitochondrial damage extends beyond a critical threshold, the quality control mechanisms of fission are initiated. Both ER-mitochondria interactions (Friedman et al., 2011) and the cytosolic protein dynamin-related protein 1 (Drp1) (Chen et al., 2003; Cribbs and Strack, 2009) are conserved features of mitochondrial fission. ER-mitochondria contact points mark the location of mitochondrial division where ER tubules physically wrap around and constrict the mitochondria, presumably to a diameter comparable to the Drp1 helices (Ingerman et al., 2005; Friedman et al., 2011). After ER constriction and upon activation, Drp1 translocates to and multimerizes around the OMM, where it pinches and severs both the IMM and OMM (Legesse-Miller et al., 2003; Lee et al., 2004; Zhu et al., 2004). Fission functions to isolate damaged components of mitochondria by segregating the damaged components of the organelle. After fission, the healthy mitochondrion is able to reincorporate into the network while the damaged mitochondrion is inhibited from reincorporation by a reduction in expression of fusion mediators, such as Opa1. This allows the damaged mitochondria to then be packaged into autophagic vacuoles that are delivered to the lysosome for disposal by the autophagic mechanism of mitophagy (Archer, 2013).

Severely damaged or superfluous mitochondria are degraded by the mitophagy—a specialized form of autophagy that targets individual mitochondria. During mitophagy, whole mitochondria are sequestered into autophagosomes and sent to lysosomes for degradation. Mitophagy is regulated by both the mitochondrial phosphatase and tensin homolog (PTEN)-induced kinase 1 (Pink1) and the cytosolic E3 ubiquitin ligase Parkin (Pellegrino and Haynes, 2015). In healthy mitochondria, expression of Pink1 is repressed by its transport into the IMM and subsequent degradation (Yamano and Youle, 2013; Thomas et al., 2014). However, in damaged mitochondria, Pink1 fails to be imported into the IMM, and instead integrates into the OMM (Geisler et al., 2010; Narendra et al., 2010) with its kinase domain exposed to the cytosol (Zhou et al., 2008), which subsequently recruits Parkin from the cytosol (Geisler et al., 2010; Narendra et al., 2010). Once recruited, Parkin ubiquitinates proteins on the OMM, targeting the mitochondrion for autophagic elimination (Narendra et al., 2008). This mitophagy pathway is also intimately connected with mitochondrial mobility. A major component of mitochondrial transport is mitochondria Rho-GTPase (Miro), a mitochondrial adaptor protein that attaches kinesin motors to the surface of mitochondria. Pink1 and Parkin associate with Miro upon depolarization of the mitochondrial membrane potential, triggering Pink1 to phosphorylate the mitochondrial adaptor protein, subsequently resulting in Parkin-dependent proteosomal degradation of Miro. Degradation of Miro causes the mitochondrion and the kinesin motor complex to separate, arresting mitochondrial motility (Wang et al., 2011). Arrest of mitochondrial motility, like degradation of mitochondrial fusion proteins, potentially functions to quarantine damaged mitochondria from reincorporating into the mitochondrial network, since static mitochondria are less prone to undergo fusion with other mitochondria (Twig et al., 2010). Homeostasis of the mitochondrial network as well as the proper functionality of the mitochondria is dependent on the cooperation of these cellular functions.

The mitochondrial population must be sustained in order to maintain cellular bioenergetic homeostasis and ensure cellular energy demands are being fulfilled. The plasticity of mitochondrial function and structure is an essential feature to maintaining cellular homeostasis, and indeed, changes in mitochondrial mass have been documented in both health and disease. For example, mitochondrial biogenesis increases in muscle cells upon exercise (Holloszy, 1967). Conversely, research has shown that as mammals age, there is a general decline in both mitochondrial mass and function (Yan and Sohal, 1998; Liu et al., 2002; Chistiakov et al., 2014). There is a wide range of clinical conditions that result from mitochondrial dysfunction, including muscular disorders, cardiomyopathy, diabetes, cancer, deafness, lactic acidosis, and skeletal myopathy (Vafai and Mootha, 2012). In addition, studies show that 1 in every 5000 individuals is affected by a mitochondrial disease (Pfeffer et al., 2012). Mitochondrial dysfunction can affect cell signaling through ROS and metabolites, and can interrupt the intimate physical connections between the mitochondria and other organelles (e.g., ER, etc.). Additionally, mitochondrial dysfunction has severe consequences on the bioenergetics of the cell. Understanding the complex responsibility mitochondria carry in the biology of cell processes and how mitochondrial dysfunction leads to disease can help target specific cellular mechanisms for the treatment and/or prevention of disease.

**Figure 1:**
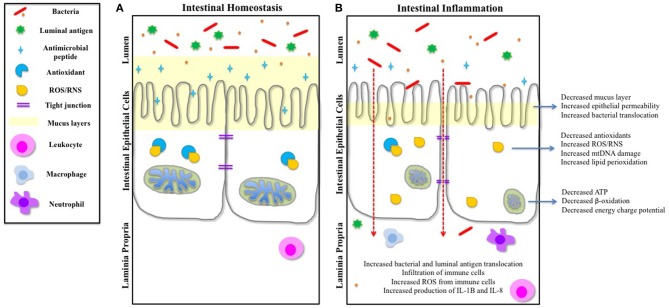
**IEC function and intestinal homeostasis can be influenced by mitochondrial dysfunction. (A)** During intestinal homeostasis, goblet cells produce a healthy mucus layer that protects the IECs from the contents of the lumen, and Paneth cells produce and release antimicrobial peptides to protect IECs. Mitochondria are dense and contain well-formed cristae. The tight junctions inhibit translocation of luminal antigens across the epithelial barrier. Any basal ROS produced is negated by cellular antioxidants. Leukocytes survey the laminia propria for threats. **(B)** Studies have shown that during the inflammatory conditions of IBD, the mucus layer is reduced and production of antimicrobial peptides is decreased, exposing the intestinal epithelium to the intestinal microbiota and luminal antigens. Mitochondria are swollen and abnormal, and cristae are irregular, resulting in a reduction in ATP production and an increase in ROS. Cellular antioxidants are also decreased, causing a buildup of cellular ROS. There is an increase in epithelial permeability (both transcellular and paracellular) and translocation of bacteria and luminal antigens. This results in an infiltration of immune cells, which also causes an increase of ROS. Both IL-8 and IL-1B are released by immune cells, and immune cell-bacterial interactions further instigates the release of pro-inflammatory mediators, which can feedback onto the IECs and influence other cellular components of the intestinal epithelium.

A variety of conditions and stimuli can alter mitochondrial function. Any disruption of mitochondrial performance can affect overall cellular function and eventually tissue/organ function. Here we review data supporting a role for mitochondrial dysfunction in the development and/or progression of IBD (Figure 1). Although a causative role of mitochondrial stress in IBD has not yet been established, the current literature would support a key correlation between mitochondrial function and intestinal inflammation. While we discuss several potential mechanisms by which mitochondrial function may impact disease, it is important to note that all of these processes are interconnected themselves. For example, an alteration in mitochondrial morphology can lead to defective mitochondrial function and communication, build-up of ROS, and activation of the inflammasome, potentially culminating in a disruption of the intestinal barrier, increased permeability, and ultimately intestinal inflammation. Additionally, some of the mechanistic links of mitochondrial dysfunction discussed are more strongly supported by scientific and clinical research (e.g., ROS generation, NLRP3 inflammasome, and autophagy). Nonetheless, it is important to understand how any alteration in the multifaceted functionality of the mitochondrion may contribute to the initiation and propagation of an inflammatory insult.

## Mitochondrial dysfunction in IBD

### Form and function

Mitochondrial form and function are intimately connected in normal cells. The mitochondria are compartmentalized organelles surrounded by two protein-containing phospholipid bilayers. The OMM encloses the entire organelle, and in conjunction with the IMM, separates the IMS and matrix compartments (Perkins and Frey, 2000; Strauss et al., 2008). Both the OMM and IMM contain translocases that function as mitochondrial protein entry ports, directing proteins to the correct subcompartment. The OMM also serves as a central signaling hub for several signal transduction pathways in the cell (Nunnari and Suomalainen, 2012). For example, innate antiviral immunity modulated by mitochondria is dependent upon mitochondrial antiviral signaling (MAVS), an OMM adaptor protein (Seth et al., 2005; Koshiba, 2013); and mitochondria-associated membranes (MAMs) are areas on the OMM where the ER and mitochondrion physically and functionally interact (Pizzo and Pozzan, 2007). The OMM is freely permeable to small molecules, and as such, the intermembrane space contains the same concentration of small molecules (e.g., ions, sugars, etc.) that are also present in the cytosol (Stowe and Camara, 2009). Cytochrome *c*, a protein that is integral to respiration as well as the induction of apoptosis, localizes to the intermembrane space (Koehler et al., 2006; Webb et al., 2006). The IMM, an impermeable membrane that allows for the gated exchanged of metabolites and proteins and undergoes intense folding into cristae to increase the membrane surface area, encloses the matrix compartment. The IMM facilitates lipid trafficking and respiratory complex formation (five complexes in mammals) (Perkins and Frey, 2000; Strauss et al., 2008), which are involved in oxidative phosphorylation and ATP production (Arco and Satrústegui, 2005). The matrix houses numerous copies of the circular mitochondrial genome as well as the machinery needed for its replication, transcription, and subsequent translation of the encoded proteins. Additionally within the matrix is a diverse set of enzymes required for cellular metabolic processes, including fatty-acid synthesis, the Tricarboxylic Acid Cycle (TCA), heme-synthesis, and iron-sulfur cluster formation (Ryan and Hoogenraad, 2007).

There are five primary functions of the mitochondria that are pivotal to mitochondrial form-function dynamics. First, mitochondrial biogenesis regulates the mitochondrial population in order to meet the energy requirements of the cell (Archer, 2013). Second, the cell maintains the health of mitochondria through the process of mitophagy, which eliminates damaged, depolarized mitochondria via lysosomal vacuoles. Mitophagy is facilitated by both the process of fission, which isolates depolarized mitochondria and suppresses fusion mediators, and by inhibiting the reorganization of the damaged mitochondria back into the network (Archer, 2013). Third, mitochondria are mobile organelles that transverse a dynamic network of dynein and kinesin within the cytosol (Wang et al., 2011). While the relationship between mitochondrial mobility and form and function is not clear, the dynein/dynactin complex is known to regulate the process of fission by recruiting Drp1 to the OMM (Varadi et al., 2004). Fourth, mitochondria are important oxygen-sensing beacons in the cell, and the initial steps in the mechanism of redox signaling depend upon mitochondrial dynamics (Marsboom et al., 2012; Hong et al., 2013). Lastly, mitochondria are linked to the ER through MAMs, enabling these two organelles to communicate through calcium signaling, which has affects on oxidative metabolism and apoptosis (Szabadkai et al., 2004; Denton, 2009; Patergnani et al., 2011). Hence, a minor perturbation in mitochondrial structure or function can lead to mitochondrial dysfunction, which can have deleterious effects on the cell.

Supporting the importance of mitochondrial form and function, enterocytes isolated from patients with IBD have been reported to exhibit swollen mitochondria with irregular cristae (Delpre et al., 1989; Söderholm et al., 2002b; Nazli et al., 2004). Abnormal mitochondrial structure is also seen in intestinal epithelial cells (IECs) from mice subjected to experimental models of colitis (Rodenburg et al., 2008). These morphological changes are suggestive of cellular stress and bioenergetic failure. Indeed, patients with IBD have reduced ATP levels within the intestine (Roediger, 1980a; Kameyama et al., 1984; Schürmann et al., 1999). As would be expected, morphological changes in mitochondria have been shown to result in deficiencies in the β-oxidation of short-chain fatty acids (SCFA) (Halestrap and Dunlop, 1986). It remains unclear, however whether observed changes in mitochondrial structure come as a result of disease or whether they may play a role in the pathogenesis of inflammation.

### Intestinal epithelial barrier function

Numerous cellular processes are dependent upon healthy mitochondria for an adequate energy supply. The intestinal mucosa of IBD patients has been demonstrated to be in a state of energy deficiency characterized by low ATP levels and low energy charge potential, (Roediger, 1980a; Kameyama et al., 1984; Söderholm et al., 2002a), calling into question the functionality of this organelle during disease. Indeed, the colonic epithelial cells of patients with ulcerative colitis exhibit mitochondrial alterations before other ultrastructural abnormalities in the epithelium are apparent and before the onset of mucosal inflammation (Delpre et al., 1989; Hsieh et al., 2006). The integrity of the intestinal epithelium, tight junction maintenance, and β-oxidation are key cellular processes within the intestinal epithelium that are not only dependent upon properly functioning mitochondria, but are also known to be altered in animal models of intestinal inflammation and in humans with IBD.

#### Intestinal epithelial cells (IECs)

It is known that IBD is a multifactorial disease, involving the interplay of immune dysregulation, genetic susceptibility, environmental factors, and microbial dysbiosis. The intestinal epithelium comprises the interface between these factors, and thus, may play a vital role in governing this interplay. A key feature of IBD is recurrent damage of the intestinal epithelium concomitant with disruption of the intestinal barrier function (Roda et al., 2010; Henderson et al., 2011; Salim and Söderholm, 2011). The intestinal epithelium is the host's defensive barrier against the luminal microenvironment with discriminatory absorption of nutrients and antigen permeability. The intestinal epithelium is in a constant state of turnover, renewing every 4–5 days and necessitating a considerable supply of energy. The epithelium is comprised of a single layer of different subtypes of IECs, including absorptive enterocytes, mucus-producing goblet cells, enteroendocrine cells, and defensin-producing Paneth cells—all of which differentiate from intestinal epithelial Lgr5+ stem cells (Gibson et al., 1996; Crosnier et al., 2006; van der Flier and Clevers, 2009). The intestinal stem cells are believed to undergo asymmetric division to give rise to transit amplifying (TA) progenitor cells, which are rapidly cycling cells that amplify the progeny of the stem cells, undergoing a limited number of divisions before terminally differentiating into a mature cell lineage and being sloughed off at the villus tip. The cellular structure of the epithelium is organized in space, such that the proliferating stem cells are buried in the crypts and the differentiated mature cells migrate up the surface of the villi (Gibson et al., 1996; Crosnier et al., 2006; van der Flier and Clevers, 2009). Each subset of IECs serves a unique purpose within the epithelium, yet all are critical for intestinal homeostasis and modulating the crosstalk between the microbial community and the circulating immune cells (van der Flier and Clevers, 2009; Noah et al., 2011; Dupaul-Chicoine et al., 2013). Consequently, dysregulation of IEC differentiation has serious effects on the pathogenesis of IBD, and several genes for IEC differentiation have been shown to be perversely expressed in the setting of inflammation (Ahn et al., 2008; Zheng et al., 2011; Coskun et al., 2012). Indeed, depletion of mucus and goblet cells is a characteristic of patients with ulcerative colitis (Jass and Walsh, 2001; Danese and Fiocchi, 2011). Muc2-deficient mice, which lack the gene encoding the major component of mucin, spontaneously develop colitis (Van der Sluis et al., 2006). Likewise, several IBD susceptibility genes are associated with Paneth cell dysfunction. For example, the *Nod2* risk allele for Crohn's disease is associated with a decrease in α-defensin production by Paneth cells in humans (Wehkamp et al., 2004, 2005), and NOD2-deficient mice also exhibit a decrease in α-defensin production (Kobayashi et al., 2005). Paneth cell dysfunction in both humans and mice is also associated with autophagy related 16-like 1 (ATG16L1) (Cadwell et al., 2008, 2009) and X-box binding protein 1 (XBP1) (Kaser et al., 2008), both of which are associated with increased risk of Crohn's disease (Rioux et al., 2007; Kaser et al., 2008). Adolf et al. has shown that by deleting both ATG16L1 and XBP1, mice develop spontaneous CD-like ileitis, which may be a consequence of Paneth cell dysfunction (Adolph et al., 2013). Furthermore, mice lacking caspase-8, a cysteine protease involved in mediating cellular apoptosis, had reduced numbers of goblet cells, no Paneth cells, and also spontaneously developed ileitis (Günther et al., 2011). Thus, defects in intestinal epithelial homeostasis results in an inadequate intestinal barrier defense, which may allow luminal antigens and/or microbes to interact with or violate the intestinal epithelium and consequently cause inflammation (Gersemann et al., 2011). However, the role of mitochondrial dysfunction during IEC differentiation needs to be further evaluated in order to understand the role it may play in the development of intestinal inflammation. Interestingly, Bär et al. demonstrated that altered mitochondrial oxidative phosphorylation activity influences intestinal inflammation in models of experimental colitis using strains of conplastic mice, which have identical nuclear genomes but diverse mitochondrial genomes (Bär et al., 2013). Two strains of conplastic mice, which had increased concentrations of intestinal ATP and augmented oxidative phosphorylation complex activity, were protected from dextran sodium sulfate (DSS)- and trinitrobenzene sulfonate (TNBS)-induced colitis. These mice also had increased proliferation of enterocytes, suggesting that increased intestinal ATP levels (which were due to mtDNA polymorphisms) caused a surge in the turnover rate of the intestinal epithelium—a process that is central to the renewal of the epithelium after exposure to harsh conditions and noxious provocations, such as DSS and TNBS (Bär et al., 2013). This study suggests that increased regeneration of the intestinal epithelium (by means of increased mitochondrial function) is a key factor in combating intestinal inflammation. Indeed, recent clinical evidence has demonstrated that complete mucosal healing is associated with long-term remissions and decreased risk of operative intervention in IBD patients. Mucosal healing also results in the improved mitochondrial structure in the IECs of patients with ulcerative colitis (Fratila and Craciun, 2010). Aside from the providing energy supplies for cell differentiation, there is accumulating evidence that mitochondria play additional roles in cellular differentiation (Maeda and Chida, 2013; Xu et al., 2013; Weinberg et al., 2015). Thus, it is possible that mitochondrial dysfunction could impact IEC differentiation either through energy production or signaling networks, adversely affecting the integrity of the epithelial cell barrier and potentially influencing the development of disease

#### Tight junctions

The capacity of the intestinal epithelium to function as a physical, protective barrier is dependent upon tight junctions (TJs), which seal the paracellular space between epithelial cells and polarize the cell membrane. TJs contribute to the integrity of the gut barrier by controlling paracellular permeability and barrier competence of the intestinal epithelium as well as contributing to mucus layer production and infection control (Peterson and Artis, 2014). There are studies that provide a strong link between the development of IBD and altered expression and structural modifications of TJs. Indeed, evidence shows an association between aberrant intestinal permeability and intestinal mucosal inflammation in IBD (Schmitz et al., 1999; Heller et al., 2005; Zeissig et al., 2007). Reports have correlated increased intestinal permeability in first-degree relatives of patients with IBD, and interestingly, studies have also demonstrated that the spouses of patients with IBD can experience increased gut permeability (May et al., 1993; Söderholm et al., 1999; Breslin et al., 2001; Thjodleifsson et al., 2003). Maintenance of TJ integrity is energy-dependent, and it is not surprising that disruption of the barrier by toxins, pathogens, or noxious stimuli can be initiated by damaged mitochondria (Dickman et al., 2000; He et al., 2000). Certain insults, such as NSAID exposure, are known to disrupt the structure and function of mitochondria, and at least transiently, increase gut permeability (Somasundaram et al., 1997, 2000; Söderholm et al., 1999; Zamora et al., 1999; Basivireddy et al., 2002). Additionally, it has been reported that some patients with Crohn's disease develop immune reactivity against components of their gut microbiome (Pirzer et al., 1991; Duchmann et al., 1996). Consistent with these reports, Nazli et al. demonstrated that treating a cell monolayer with dinitrophenol (an oxidative phosphorylation uncoupler) resulted in cellular internalization of a non-invasive strain of *Escherichia coli*. From this, the authors hypothesized that under metabolic stress resulting from mitochondrial dysfunction, the enteric epithelium loses its ability to distinguish between commensals and pathogens, and as a result, begins internalizing commensal organisms, which can lead to an exacerbated intestinal inflammatory response (Nazli et al., 2004). The mechanism behind developing reactivity to one's own microbiota is not understood, and more research is needed to delineate the role of metabolic stress (e.g., energy deprivation as a result of decreased mitochondrial function) in this process. Studies do suggest that both mitochondrial dysfunction (Lewis and McKay, 2009) and increased gut permeability (De-Souza and Greene, 2005; Deitch et al., 2006) affect the overall competence of the intestinal epithelial barrier, but the stimuli that initiates either process is not known. Nonetheless, these studies lend credence to the implication of epithelial mitochondrial dysfunction as a predisposing factor for an increase in gut epithelial permeability and a loss of gut barrier function, resulting in intestinal inflammation.

#### β-oxidation

IBD has been suggested to involve a state of energy-deficiency, whereby oxidative metabolism is altered within IECs (Fukushima and Fiocchi, 2004; Saitoh et al., 2008). The SCFA butyrate is the preferred energy source of colonic epithelial cells (Roediger, 1980a; Hamer et al., 2008) and also plays a role in maintaining colonic mucosal health (Hamer et al., 2008). It is a natural nutrient both found in food and produced as an intestinal fermentation by-product of dietary fiber by gut bacteria (Santhanam et al., 2007). Butyrate undergoes catabolic degradation through β-oxidation in the mitochondrial matrix of colonocytes, providing over 70% of the energy demand of the colonic epithelium (Roediger, 1980b). Butyrate metabolism was demonstrated to be impaired in an animal model of colitis (Ahmad et al., 2000), and numerous studies have reported impaired metabolism in the intestinal mucosa of patients with IBD (Roediger, 1980a; Kameyama et al., 1984; Harig et al., 1989; Ramakrishna et al., 1991; Chapman et al., 1994). Similarly, intestinal mucosal inflammation results when butyrate oxidation is inhibited in experimental animals (Roediger and Nance, 1986). Santhanam et al. showed that the mitochondrial acetoacetyl CoA thiolase, which catalyzes the critical last step in butyrate oxidation, was significantly impaired in the colonic mucosa of patients with ulcerative colitis. Furthermore, they conclude that an increase in mitochondrial ROS may trigger this enzymatic defect (Santhanam et al., 2007). Polymorphisms in *SLC22A5*, the gene that encodes for the carnitine transporter OCTN2, is a known risk factor for IBD (Barrett et al., 2008; Singh et al., 2009a). IECs utilize carnitine as a transporter of long-chain fatty acids into the mitochondria for β-oxidation (Rinaldo et al., 2002). Furthermore, genetic ablation of OCTN2 as well as pharmacologic inhibition of intestinal fatty acid β-oxidation results in murine experimental colitis (Roediger and Nance, 1986; Shekhawat et al., 2007). Studies involving the treatment of epithelia cells with dinitrophenol to induce mitochondrial stress resulted in decreased transepithelial resistance and increased bacterial translocation (Lewis et al., 2010)—both of which are features of gut barrier dysfunction. Thus, defective β-oxidation in the mitochondria has deleterious effects beyond energy requirements. Likewise, a dysfunctional gut microbiome or a poor diet may also result in a decrease of butyrate metabolism in the colonic epithelium. Enhanced production of butyrate may potentially benefit the colonic epithelial cells by stimulating an enhancement in cellular homeostasis, including antioxidant and anti-inflammatory roles as well as protective gut-barrier functions.

### Reactive oxygen species (ROS) and reactive nitrogen species (RNS)

Oxidative stress within the intestinal epithelium is thought to play a key role in the pathogenesis of intestinal inflammation (Grisham, 1994; Elson et al., 1995; Conner et al., 1996). Although ROS and RNS are important signaling intermediates involved in a variety of homeostatic molecular pathways (Brown and Griendling, 2009; Gillespie et al., 2009), excessive oxidative stress can provoke cellular damage through the oxidation of proteins, lipids, and DNA, altering their biological functions and potentiating cell death (Andersen, 2004). At baseline, the deleterious effects of ROS generation are negated by a plethora of endogenous antioxidants (Haddad, 2002; Gillespie et al., 2009). The intestinal lumen and epithelium are continuously exposed to noxious stimuli, such as ingested nutrients, local microbes or infections, gastric acid production, and periods of ischemia/reperfusion that have the potential to stimulate the generation oxygen and nitrogen radicals (Parks et al., 1988; Parks, 1989; Young and Woodside, 2001; Sánchez et al., 2002; Biswas et al., 2003; Mazalli and Bragagnolo, 2009). Additionally, the infiltration of leukocytes, monocytes, and neutrophils during inflammation can further enhance intestinal ROS production through both respiratory burst enzymes and prostaglandin and leukotriene metabolism (Babbs, 1992). Several studies have demonstrated increased ROS/RNS levels within the intestinal epithelium of patients with IBD (Kruidenier and Verspaget, 2002; Pravda, 2005; Rezaie et al., 2007) and in murine models of experimental colitis (Girgin et al., 1999; Tham et al., 2002; Narushima et al., 2003; Sundaram et al., 2003; Oz et al., 2005; Siddiqui et al., 2006; dos Reis et al., 2009; Kajiya et al., 2009; Abdolghaffari et al., 2010; Yao et al., 2010; Lenoir et al., 2011; Ock et al., 2011; Sengül et al., 2011; Borrelli et al., 2013; Arab et al., 2014). High concentrations of oxidized molecules have also been measured in the plasma, serum, exhaled air, and saliva of patients with IBD (Tüzün et al., 2002; Rezaie et al., 2006). Others have shown a positive correlation between oxidative stress and disease severity, suggesting a role in the development and potentiation of inflammation (Rachmilewitz et al., 1995, 1998; Herulf et al., 1998).

In addition to changes in the generation of reactive species, several studies have shown an overall reduction in endogenous antioxidants, such as ascorbate, β-carotene, α-tocopherol, and reduced glutathione, in patients with IBD (Buffinton and Doe, 1995; McKenzie et al., 1996; Schorah, 1998; Sido et al., 1998; Geerling et al., 2000). Interestingly, mice lacking an important antioxidant enzyme, glutathione peroxidase, spontaneously develop symptoms and histologic features similar to those in IBD patients (Esworthy et al., 2001). Moreover, in a murine DSS-induced colitis model, mice subjected to DSS exhibited diminished blood levels of reduced glutathione, which were restored to normal after treatment with various antioxidants (Oz et al., 2005). Levels of catalase, glutathione peroxidase, and superoxide dismutase at baseline have been shown to be lower in the human colonic mucosa, submucosa, and serosa as compared to the human liver (Grisham et al., 1990; Mulder et al., 1991; Buffinton and Doe, 1995) and small intestine (Blau et al., 1999), suggesting a limited capacity to combat oxidative stress in the setting of inflammation. Furthermore, treatment with a mitochondria-targeted antioxidant, MitQ, reduced mitochondrial ROS and protected against experimental colitis in mice subjected to DSS (Dashdorj et al., 2013). Likewise, Wang et al. reported that mitochondrial superoxide was the principal initiator of internalization and transcytosis of a commensal microbes across metabolically stressed epithelium in cell lines and human colonic tissue analyzed *ex vivo*, and that treatment with mitochondrially-targeted antioxidants countered this epithelial barrier defect (Wang et al., 2014). However, it is not yet fully understood if the correlation between ROS/RNS and IBD predicts an actual etiologic relationship for oxidative stress in intestinal inflammation, or if reactive molecular species are merely a consequence of the inflammatory process.

Oxidative stress is thought to exert deleterious effects largely though direct DNA damage and lipid oxidation. Multiple studies have reported increased oxidative DNA damage in the blood and mucosa of patients with IBD (Lih-Brody et al., 1996; D'Incà et al., 2004; Dincer et al., 2007). It has been suggested that these changes may contribute to the increased susceptibility to colorectal cancer that is seen in IBD later in life (Persson et al., 1994; Canavan et al., 2006; Grivennikov, 2013). Colonic mucosal biopsies and plasma from IBD patients also demonstrate an increase in lipid peroxidation products, implying increased ROS production (Pereira et al., 2015). Mice deficient in the antioxidant not only show evidence of increased lipid peroxidation products in both the colon and ileum, but also spontaneously develop colitis (Esworthy et al., 2001), indicating that the ability to combat the oxidative degradation of lipids may be critical in maintaining intestinal homeostasis.

Mitochondria are the most abundant source of ROS in the cell (Beltrán et al., 2010). Under healthy cellular conditions, low levels of ROS are generated and neutralized by the endogenous antioxidant machinery (Haddad, 2002; Gillespie et al., 2009). However, when mitochondria are destabilized by damage or mutations, excessive oxidative stress may result, leading to a reduction of ATP, inhibition of the respiratory chain, and mtDNA damage (Du et al., 1998). Prolonged oxidative stress reduces mitochondrial bioenergetics and homeostasis, promoting cellular damage and ultimately cell death (Scherz-Shouval and Elazar, 2007; Chen and Gibson, 2008). Recent studies have proposed mitochondria as significant cellular drivers and mediators of the inflammatory process (Lee and Hüttemann, 2014). The mitochondria are also a major target of the deleterious effects of oxidative stress, but little is known about how this may lead to inflammation. Understanding the role of mitochondrially-derived ROS in the pathogenesis of IBD may offer key insights into the initiation and propagation of disease.

### NLRP3 inflammasome

Mitochondria are capable of regulating the pro-inflammatory response of the cell through activation of a molecular complex known as the inflammasome. The inflammasome is a multi-protein, caspase-1 activating complex. NLRP3 (NLR family, pyrin domain containing 3) has emerged as critical regulator of intestinal homeostasis (Davis et al., 2014). Formation of the NLRP3 inflammasome is activated by pathogen-associated molecular patterns (PAMPs) as well as damage-associated molecular patterns (DAMPs) that signify cellular stress, such as extracellular ATP, mtDNA, and ROS (Martinon et al., 2002; López-Armada et al., 2013). Once activated, NLRP3 associates with the adaptor molecule ASC (apoptosis-associated speck-like protein), which contains a caspase recruitment domain (CARD). The associated NLRP3-ASC complex oligomerizes and recruits procapase-1, resulting in formation of the active inflammasome, which in turn causes autocleavage of caspase-1 and release of activated inflammatory cytokines IL-8 and IL-1β (Tschopp, 2011; Zitvogel et al., 2012). Data from murine experimental colitis models and human intestinal specimens reveal that elevated expression of IL-8 and IL-1β is central to the pathogenesis of IBD (Sartor, 1994; Ishiguro, 1999; Monteleone et al., 1999; Kwon et al., 2005; Maeda et al., 2005; Ishihara et al., 2013). IL-8 induces expression of IL-1β and other pro-inflammatory cytokines (Kim et al., 2010), which results in intense intestinal inflammation. IL-1β has been shown to increase gut permeability, which then allows for increased bacterial translocation (Al-Sadi et al., 2012). Furthermore, the secretion of biologically active IL-8 and IL-1β is mediated by caspase-1, which has been reported to play a role in DSS-induced colitis (Siegmund et al., 2001). Bauer et al. demonstrate that DSS induces activation of caspase-1 through NLRP3 inflammasome activation. They further show that NLRP3-deficient mice are protected from DSS-induced colitis, exhibiting a significantly reduced production of pro-inflammatory cytokines as well as improved clinical assessments and histological scores (Bauer et al., 2010). Additionally, polymorphisms in the *Nlrp3* gene are associated with an increased susceptibility to Crohn's disease (Villani et al., 2009). Schoultz et al. has reported that polymorphisms in the genes encoding both *Nlrp3* and *Card8*, a potent component of the NLRP3 inflammasome, confer increased susceptibility to developing Crohn's disease in Swedish men (Schoultz et al., 2009).

The exact mechanism of NLRP3 inflammasome activation in IBD is not yet known. Several studies have revealed that the NLRP3 inflammasome is involved in murine experimental colitis, and that stimulation of the inflammasome was modulated by mitochondrial ROS (Shimada et al., 2012). However, there remains controversy about the source of ROS that activates intestinal inflammation. Mitochondria are a major, but not the only source of ROS production in the cell. Dashdorj et al. recently published a study implicating mitochondrial ROS as the instigator of inflammation activation (Dashdorj et al., 2013). Consistent with this, other studies have demonstrated that inflammasome activation occurs in mice deficient in nicotinamide adenine dinucleotide phosphate (NADPH) oxidase subunits—a membrane-bound enzymatic complex that functions to generate superoxide, a type of ROS. Additionally, stimulation of the inflammasome also occurs in the mononuclear phagocytes from patients with chronic granulomatous disease, a condition that stems from mutations in the NADPH oxidase subunits (Meissner et al., 2010). Thus, it seems likely that mitochondrial ROS production plays a key role in the intestinal inflammation associated with IBD. However, more research is needed to delineate the extent to which the mitochondria and its concomitant ROS and oxidized mtDNA are involved in the development and progression of intestinal inflammation. Recent studies have begun to appreciate the communication that occurs between mitochondria and pathogen recognition receptors (PRRs) (West et al., 2011). It is interesting to consider the role this communication may play in maintaining immune-microbial homeostasis in the intestinal tract, and how mitochondrial dysfunction may affect the development of intestinal inflammation. Studying the molecular behavior of the inflammasome and its downstream effectors in IBD should add important insights into the mechanistic pathways relevant to the pathogenesis and treatment of disease.

### Mitochondrial communication

Sustaining the mitochondrial population in the cell is central to maintaining cellular bioenergetic homeostasis. Mitochondria have a dedicated repertoire of quality control machinery dedicated to maintaining protein-folding homeostasis. They are composed of four compartments, each of which is a separate protein-folding environment that must be maintained for proper function. Chaperone proteins are localized in the matrix and are required for protein import and promote proper protein folding. In addition to matrix-localized chaperones, there are proteases located in the matrix and IMM, which function to recognize and degrade proteins that fail to fold or assemble properly (Tatsuta and Langer, 2008). Only approximately 10% of the proteins that comprise the electron transport chain are encoded by the mitochondria. The remainder are encoded by the nucleus, translated in the cytosol, and then transported into the mitochondria, where they subsequently assemble into stoichiometric complexes with mitochondrial-encoded proteins (Haynes and Ron, 2010). Thus, it is apparent how cellular stress, such as excessive ROS, mutated proteins, or environmental stress, can negatively affect the protein-folding capacity of the mitochondria, resulting in an accumulation of misfolded proteins or misassembled protein complexes (Ron and Walter, 2007; Ryan and Hoogenraad, 2007). The cell has evolved several quality control pathways to monitor mitochondrial homeostasis and prevent mitochondrial dysfunction. One of these pathways, the mitochondrial unfolded protein response (UPR^mt^), is a protective response that fosters survival during times of mitochondrial dysfunction or stress by functioning to lessen proteotoxic stress and re-establish protein homeostasis by increasing the population of mitochondrial quality control proteases and chaperones. The UPR^mt^ is a mitochondrial-nuclear cross-talk pathway that, upon communication of unfolded protein stress, activates the transcription factor C/EBP homologous protein (CHOP) (Papa and Germain, 2014), which in turn induces expression of UPR^mt^-responsive genes (Haynes et al., 2007; Horibe and Hoogenraad, 2007; Baker et al., 2011). It this thought that the UPR^mt^ functions to stabilize and promote the recovery of those mitochondria that are not beyond repair, whereas those organelles that are not salvageable are targeted for mitophagy (Haynes et al., 2013).

Numerous diseases, particularly metabolic and neurogenerative diseases are associated with mitochondrial dysfunction. Some diseases, such as spastic paraplegia, stem directly from mutations that impair mitochondrial function and homeostasis (Casari et al., 1998; Hansen et al., 2002). Most diseases that are associated with mitochondrial dysfunction, though, display characteristics, such as an accrual of mtDNA mutations, augmented ROS generation, and a reduction in ATP output (Haynes and Ron, 2010). All of these features secondarily affect the protein-folding environment of the mitochondria and are common to IBD. However, the exact role UPR^mt^ plays in IBD is just beginning to be uncovered. Rath et al. has recently demonstrated that UPR^mt^ signaling interfaces with the ER unfolded protein response (UPR^ER^) pathway via double-stranded-RNA-activated protein kinase (PKR). Additionally, IECs were unable to activate *cpn60*, an UPR^mt^ target gene, in PKR-deficient mice subjected to DSS, resulting in resistance to DSS-induced colitis (Rath et al., 2012). This study suggests that the UPR^mt^ has a role in the pathogenesis of IBD, and since it seems that PKR integrates UPR^mt^ signaling into UPR^ER^, then both mitochondrial and ER protein homeostatic responses might contribute to intestinal inflammation.

The UPR^mt^ is similar to the well-known UPR^ER^. While both processes contain their own set of chaperones and proteases, and seem to be two distinct pathways, both signaling pathways share the transcription factor CHOP (Horibe and Hoogenraad, 2007) and converge together at PKR (Rath et al., 2012). Moreover, the mitochondria and ER are not only functionally connected, but also physically connected via MAMs, which play a role in calcium homeostasis and lipid biosynthesis. Calcium release at MAMs may advise mitochondria to future apoptotic events (Szabadkai et al., 2004; Denton, 2009; Patergnani et al., 2011). Accumulation of misfolded proteins in the ER has been suggested to contribute to the development of IBD (Kaser and Blumberg, 2009), and various UPR^ER^ modulators have been correlated to the pathogenesis of IBD (Maloy and Powrie, 2011). Likewise, patients with active IBD normally express augmented ER stress markers in the epithelium of ileum and/or colon (Hu et al., 2007; Shkoda et al., 2007; Heazlewood et al., 2008; Kaser et al., 2008). Furthermore, mice with IEC-specific expression of a dysfunctional UPR^ER^ signaling protein displayed fragmented ER and deteriorated mitochondria (Cao et al., 2014), implying both ER and mitochondrial dysfunction. Both mitochondrial stress and ER stress have been implicated in a set of diseases associated with mitochondrial dysfunction (Fukushima and Fiocchi, 2004; Ozcan et al., 2004; Zhang and Kaufman, 2008; Lim et al., 2009; Haga et al., 2010; Rath and Haller, 2011). It is not known if mitochondrial dysfunction is a result of ER stress and dysfunction; or if a separate, external signal (e.g., diet, microbiome, ROS, etc.) damages the mitochondria, which then consequently, influences the functionality of the ER. It is also remarkable to note that butyrate has been shown to impact mitochondrial pathways and UPR^ER^ signaling in IECs (Fung et al., 2011; Kolar et al., 2011). Since protein homeostasis is sensitive to environmental conditions, it is attractive to speculate that a collaborative UPR (both ER and mitochondria) functions as an innate response to detect harsh changes in the fluctuating intestinal environment. Additionally, given that butyrate is a by-product of the microbial fermentation of SCFAs, it is possible that the composition of the gut microbiota (as well as other luminal antigens) may influence mitochondrial-ER signaling pathways. Nonetheless, it is imperative to consider the contributing role of mitochondria-ER communication in intestinal inflammation.

### Mitophagy and autophagy

Defective autophagy pathways have also been associated with several diseases, including IBD. Cells defective in autophagy accumulate ROS as well as deformed mitochondria (Mizushima and Klionsky, 2007; Saitoh et al., 2008). GWAS have implicated several autophagy genes, including *Atg16l1, Lrrk2*, and *Irgm* in the genetic susceptibility to Crohn's disease (Rioux et al., 2007; Barrett et al., 2008; Lees et al., 2011; Umeno et al., 2011). Additionally, previous studies demonstrate that a deficiency in *Atg16l1* results in an increased susceptibility to experimental colitis, abnormal appearance and distribution of Paneth cell granules, and altered mitochondria (Cadwell et al., 2008; Saitoh et al., 2008). Furthermore, Liu et al. showed *Irgm1*-deficient mice exhibited a higher frequency of tubular and swollen mitochondria and increased LC3-positive autophagic vacuoles (Liu et al., 2013). This is consistent with studies that report in humans IRGM localizes to the mitochondria, where it plays a role in mitophagy (Singh et al., 2010). Furthermore, a defect in either ATG16L1 or IRGM has been associated with reduced Paneth cell function, increased susceptibility to bacterial infection, and development of colitis (Cadwell et al., 2008; Saitoh et al., 2008; Liu et al., 2013).

Additionally, prohibitin 1 (PHB), a protein that is important in maintaining normal mitochondrial respiratory function, has been implicated in modulating autophagy. Kathiria et al. demonstrated that PHB regulates autophagy in IECs via intracellular ROS signaling. Moreover, diminished expression of PHB and inhibition of autophagy aggravated mitochondrial depolarization and reduced cell survival, suggesting PHB is an indicator that signals inflammatory stress to the cell, which stimulates autophagy in order to maintain cellular homeostasis and viability (Kathiria et al., 2012). PHB is primarily located on the mitochondria in IECs, and several lines of evidence imply it functions in regulating mitochondrial morphology and function (Artal-Sanz and Tavernarakis, 2009). Interestingly, PHB is decreased in patients with active IBD as well as in animals subjected to experimental colitis (Hsieh et al., 2006; Theiss et al., 2007). Restoration of PHB expression in colonic epithelial cells protected mice from experimental colitis and also exhibited antioxidant properties (Theiss et al., 2009, 2011). Recently, PHB has been shown to interact with the transcription factor STAT3 in colonic epithelial cells and mediate its downstream apoptotic effects. Interestingly, STAT3 has been shown to reside in the mitochondria where it promotes optimal electron transport chain activity, and its activity as a signal transducer has been implicated in IBD (Han et al., 2014).

Mitophagy has also been implicated in the pathogenesis of IBD by a study that revealed an association between single nucleotide polymorphisms in the gene SMAD specific E3 ubiquitin protein ligase 1 (SMURF1) and IBD (Franke et al., 2010). As a regulator of mitophagy, SMURF1 is recruited to damaged mitochondria, where it promotes degradation of the mitochondria by modulating the transport of the autophagic substrate to the autophagosome (Ni et al., 2015). SMURF1 was identified as a crucial mediator of viral autophagy and mitophagy (Orvedahl et al., 2011). However, further studies are needed in order to unravel the part mitophagy plays, beyond normal functions, in IBD pathogenesis. Taken together, these findings suggest autophagy as an important mediator of intestinal homeostasis. Further research is needed in order to delineate the mechanisms of autophagy and their role in intestinal inflammation. Likewise, the interrelation of mitochondrial dysfunction, autophagy, and IBD is still elusive.

## Conclusion

Mitochondrial function is undoubtedly crucial to the maintenance of the intestinal epithelium (Figure 1). IECs undergo a constant process of cellular turnover and, as such, necessitate a high-energy production at baseline. Aside from supplying the cell with energy, mitochondria also contribute to a plethora of cellular processes, rendering mitochondria central to cell and ultimately organ survival. There are several intestinal inflammatory diseases that involve mitochondrial dysfunction. For example, several clinically significant enteric pathogens that cause intestinal inflammation, including enteropathogenic *E. coli* (EPEC) (Nagai et al., 2005; Kozjak-Pavlovic et al., 2008), *Helicobacter pylori* (Ashktorab et al., 2004; Kozjak-Pavlovic et al., 2008), and *Salmonella typhimurium* (Hernandez et al., 2003; Layton et al., 2005), target effector proteins to the mitochondria of the host cell. Infection with *Citrobacter rodentium* has been shown to be result in a disruption in mitochondrial function and structure in mice (Ma et al., 2006). Mutations in mtDNA and reductions in cytochrome *c* oxidase activity have also been reported in human colorectal cancer (Heerdt et al., 1990; Alonso et al., 1997; Polyak et al., 1998; Payne et al., 2005; Namslauer and Brzezinski, 2009). IBD, hypothesized to be an energy-deficient disease of the intestinal epithelium, has been demonstrated to be associated with mitochondrial abnormalities of the intestinal epithelium, which occur before the onset of inflammation. Although there is no evidence for a causative association between mitochondrial dysfunction and IBD, here we provide several studies that demonstrate potential links connecting the two. A variety of stimuli and environmental conditions can perturb mitochondrial function, yet the primary stimuli of intestinal mitochondrial stress in IBD have yet to be determined. Since IBD is theorized to require two or more “hits” for the development of disease, it is not illogical to suggest that mitochondrial dysfunction in the intestinal epithelium is an integral component of the intestinal inflammatory process, potentially through effects on epithelial permeability, host-microbiota interactions, or effects on the signaling processes mitochondria are involved in. Nonetheless, the findings reviewed here suggest that the intestinal mitochondria may serve as a novel pharmacological target in the treatment and prevention of IBD, which is consistent with studies published recently using mitochondrial-targeted antioxidants to treat experimental colitis in mice (Dashdorj et al., 2013; Wang et al., 2014). Our understanding of mitochondrial dysfunction in intestinal inflammation is still in its infancy, and there are many more questions than answers. Elucidating the link between mitochondria and IBD will enable the development of new therapeutic strategies aimed at treating the cause of mitochondrial dysfunction, which may potentially prevent and/or treat disease by maintaining both mitochondrial health and homeostasis of the intestinal epithelium.

### Conflict of interest statement

The authors declare that the research was conducted in the absence of any commercial or financial relationships that could be construed as a potential conflict of interest.
